# The contribution of a novel *PHEX* gene mutation to X-linked hypophosphatemic rickets: a case report and an analysis of the gene mutation dosage effect in a rat model

**DOI:** 10.3389/fendo.2023.1251718

**Published:** 2023-12-05

**Authors:** Xiaoming Chen, Cijing Cai, Shaocong Lun, Qiuli Ye, Weiyuan Pan, Yushi Chen, Yuexuan Wu, Taoshan Feng, Faming Su, Choudi Ma, Jiaxin Luo, Meilian Liu, Guoda Ma

**Affiliations:** ^1^ Department of Endocrinology, Affiliated Hospital of Guangdong Medical University, Zhanjiang, China; ^2^ Faculty of Chinese Medicine, Macau University of Science and Technology, Avenida Wai Long, Taipa, Macau, China; ^3^ Maternal and Children’s Health Research Institute, Shunde Women and Children’s Hospital, Guangdong Medical University, Foshan, China; ^4^ Department of Traditional Chinese Medicine, Affiliated Hospital of Guangdong Medical University, Zhanjiang, China; ^5^ Department of Pulmonary Oncology, Affiliated Hospital of Guangdong Medical University, Zhanjiang, China; ^6^ Key Laboratory of Research in Maternal and Child Medicine and Birth Defects, Guangdong Medical University, Foshan, China

**Keywords:** X-linked hypophosphatemic rickets, genotype-phenotype, PHEX gene, gene mutation, FGF23

## Abstract

A Chinese family was identified to have two patients with rickets, an adult female and a male child (proband), both exhibiting signs related to X-linked hypophosphatemic rickets (XLH). Gene sequencing analysis revealed a deletion of adenine at position 1985 (c.1985delA) in the *PHEX*-encoding gene. To investigate the relationship between this mutation and the pathogenicity of XLH, as well as analyze the effects of different dosages of *PHEX* gene mutations on clinical phenotypes, we developed a rat model carrying the *PHEX* deletion mutation. The CRISPR/Cas9 gene editing technology was employed to construct the rat model with the *PHEX* gene mutation (c.1985delA). Through reproductive procedures, five genotypes of rats were obtained: female wild type (X/X), female heterozygous (-/X), female homozygous wild type (-/-), male wild type (X/Y), and male hemizygous (-/Y). The rats with different genotypes underwent analysis of growth, serum biochemical parameters, and bone microstructure. The results demonstrated the successful generation of a stable rat model inheriting the *PHEX* gene mutation. Compared to the wild-type rats, the mutant rats displayed delayed growth, shorter femurs, and significantly reduced bone mass. Among the female rats, the homozygous individuals exhibited the smallest body size, decreased bone mass, shortest femur length, and severe deformities. Moreover, the mutant rats showed significantly lower blood phosphorus concentration, elevated levels of FGF23 and alkaline phosphatase, and increased expression of phosphorus regulators. In conclusion, the XLH rat model with the *PHEX* gene mutation dosage demonstrated its impact on growth and development, serum biochemical parameters, and femoral morphology.

## 1 Introduction

XLH is the most prevalent type of hereditary hypophosphatemic rickets. It is characterized by an X-linked dominant inheritance and has an incidence rate of approximately 3.9 to 5 per 100,000 individuals (1). XLH patients commonly exhibit clinical manifestations such as progressive skeletal deformities, growth retardation, and dental hypoplasia. However, the severity of these manifestations varies greatly, particularly in terms of femoral morphology (2). Joint pain and osteoarthritis are frequent complications experienced by XLH patients due to skeletal deformities, fractures, enthesopathy, spinal stenosis, and hearing loss (3).

In 1995, an international collaborative research team identified XLH as a genetic disorder caused by loss-of-function mutations in the phosphate-regulating gene with homologies to endopeptidases on the X chromosome (*PHEX*) (4). The *PHEX* gene (NM_000444.6), located in the Xp22.2-p.22.1 region of the X chromosome, contains the full genome sequence of 219 kb and comprises 22 exons. This gene encodes a transmembrane protein with 749 amino acids, belonging to the type II integral membrane zinc-dependent endopeptidase family. Mutations in the *PHEX* gene can cause elevated levels of circulating fibroblast growth factor 23 (FGF23), but the exact mechanism is not fully understood (5).

As XLH is an X-linked dominant disorder, the manifestation of this disorder requires only one mutant allele. While the presence of a normal allele may mitigate the severity of XLH-related bone defects, the clinical phenotypes can still vary among affected individuals within the same family. Several studies have demonstrated that adult male patients tend to exhibit more severe skeletal phenotypes compared to heterozygous female patients, including lower limb deformities, short stature, and dental abnormalities (6, 7). However, there is ongoing debate regarding whether these differences are attributed to the presence of one normal allele in heterozygous female patients (6, 8–14).

In this study, we conducted an analysis of growth and development, serum biochemical parameters, expression of phosphorus regulators, and skeletal phenotypes in order to investigate the relationship between the dosage of *PHEX* mutation and clinical phenotypes using a rat model. The objective of this research is to enhance the quality of life for families with children affected by XLH through early intervention measures.

## 2 Case report

**Figure 1:**
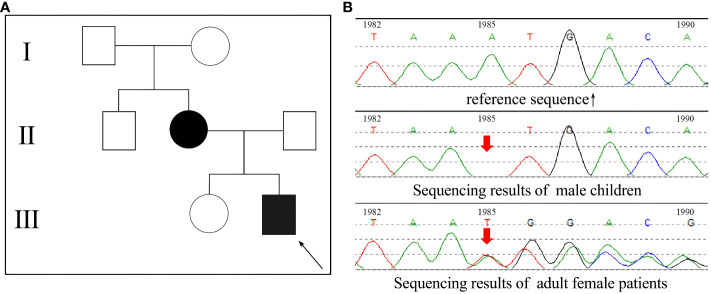
*PHEX* gene sequencing results of the patients. **(A)** The family pedigree of the patients, the proband was a male child III-2; **(B)** The gene sequencing results of the two patients.

Two cases of rickets from the same family, a mother and her child, were enrolled in this study (**Figure 1A**). The proband boy (III-2) born to a non-consanguineous couple. Both patients exhibited common clinical manifestations, including short stature, genu varus deformity, and tooth hypoplasia. The adult female patient, aged 36 and measuring 148 cm in height, reported a history of rickets during her childhood, characterized by genu varus deformity in both lower extremities and musculoskeletal pain symptoms. The male child patient, aged 8 and measuring 115 cm in height, developed genu varus deformity in both lower extremities at the age of 2, accompanied by cephalus quadratus and pigeon breast. Blood tests (serology) were performed on both patients, revealing decreased levels of phosphorus, normal levels of calcium and 25-(OH)_2_D_3_, as well as significantly elevated levels of parathyroid hormone (PTH) and alkaline phosphatase (ALP) (**Table 1**).

**Table 1:** Serum biochemical results of two patients.

	Patients		Reference range
	II-2	III-2	
P (mmol/L)	0.53	0.64	1.45~2.10
Ca (mmol/L)	2.39	2.30	2.00~2.50
PTH (pg/mL)	133.7	341.4	8.0~74.0
ALP (U/L)	723.0	810.0	51.0~250.0
25-(OH)_2_D_3_ (ng/mL)	43.2	46.6	20.0~100.0

Venous blood samples were collected from two patients, and DNA samples were extracted using column chromatography. The extraction process was conducted by KingMed (Guangzhou, China) for subsequent whole-exome gene sequencing analysis. Through this analysis, an A-base deletion at position 1985 (c.1985delA) was identified in the *PHEX*-encoding gene (**Figure 1B**). Interestingly, the c.1985del (p.Asn662fs) variation of the *PHEX* gene has been found in ClinVar, indicating its association with disease. However, research specifically investigating this genetic mutation is currently lacking. In order to determine its pathogenicity, an assessment of the gene was conducted following the guidelines provided by the American College of Medical Genetics and Genomics (ACMG). Inheritance models and published evidence were also taken into consideration. Based on the assessment, it has been determined that the c.1985del (p.Asn662fs) variation of the *PHEX* gene is a pathogenic mutation. This determination is supported by several criteria: PVS1-the patients had null variant in a gene where loss of function is a known mechanism of disease; PM2-the variant was absent from controls in gene mutation databases; PP1-the variant showed cosegregation with the disease in multiple affected family members in a gene definitively known to cause the disease; PP4-the patient’s phenotype or family history was highly specific for a disease with a single genetic etiology.

Overall, these findings indicate that the c.1985del (p.Asn662fs) variation of the *PHEX* gene is a pathogenic mutation associated with the development of low-phosphate rickets. Further research is warranted to explore its functional implications and potential therapeutic targets.

**Figure 2:**
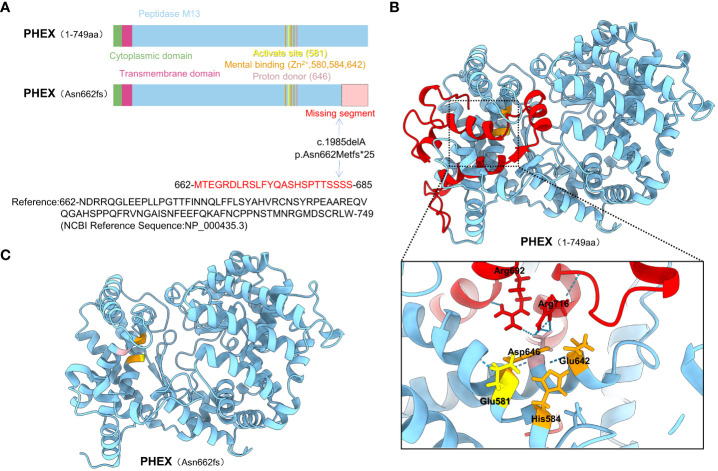
The structure prediction of the WT and mutant *PHEX* proteins. **(A)** The functional domain map of *PHEX* protein and its variant; The *PHEX* gene mutation (c.1985delA) caused a frameshift, where Asn 662 was changed to Met, followed by mistranslation, ultimately led to erroneous incorporation of 23 amino acids into a truncated protein; **(B)** The three-dimensional structure of the *PHEX* protein and the potential interactions of the missing segment with active site, metal binding sites, and proton donor site; **(C)** Prediction of three-dimensional structure of the Asn662fs mutation using AlphaFold.

This deletion leads to frameshifts, resulting in premature termination of translation and the generation of a truncated protein called p.Asn662Metfs*25. In order to investigate the underlying structural mechanism of protein dysfunction caused by the frameshift mutation (Asn662fs), we employed AlphaFold to predict the structures of both the wild-type and mutant proteins. Our analysis revealed that the frameshift mutation leads to the deletion of the C-terminal segment (663-749) within the peptidase domain. This missing segment (MS) is located in close proximity to the active site and metal ion binding sites. Moreover, it was observed that Arg692 and Arg716 within the MS region form hydrogen bonds with the proton donor site (Asp646) (**Figure 2**). Based on these findings, we speculate that the frameshift mutation disrupts the functional activity of the *PHEX* protein (**Supplementary Figure 2**).

In this study, we developed a rat model to investigate the association between deletion mutations in the *PHEX* gene and XLH. By utilizing this model, we compared the phenotypes of rats with five different genotypes to examine the correlation between *PHEX* gene mutation dosage and clinical manifestations.

## 3 Materials and methods

### 3.1 Construction of the *PHEX* gene mutation (c.1985delA) rat model and genotyping of newborn rats

Sprague Dawley (SD) rats were procured from Charles River Laboratories, and the *PHEX* gene mutation (c.1985delA) rat model was constructed by Cyagen Biosciences (Suzhou, China) using CRISPR/Cas9 gene editing technology. Progeny were genotyped by Sanger sequencing. All animal experiments were approved by the Animal Ethics Committee of the Affiliated Hospital of Guangdong Medical University and reared according to standard methods.

### 3.2 Body weight measurement and mortality statistics of rats

The body weight of rats was monitored and the mortality was calculated after the birth of newborn rats for 12 weeks; at 12 weeks of age, the body and tail morphology of rats in each group were recorded for further analysis.

### 3.3 Measurement of serum biochemical parameters

At the age of 12 weeks, the rats were anesthetized, and abdominal aortic blood samples were collected from the rats and preserved at -80°C until analysis. The phosphorus, calcium, creatinine, blood urea nitrogen, and ALP levels in blood samples were detected using Cobas 8000 automatic biochemical analyzer (Roche, Switzerland); The concentrations of FGF23 and PTH in serum were determined using enzyme-linked immunosorbent assay (ELISA) kits.

### 3.4 Quantitation of the mRNA expression

The total RNA was extracted from the left femoral shaft and kidney tissues of the rats. Real-time quantitative PCR (RT-qPCR) was performed to determine the expression of phosphorus regulators (FGF23, MEPE, and SFRP-4) and phosphorus metabolic factors (Kl, Slc34a1, and Slc34a3). GAPDH served as an internal control in both the left femoral shaft and kidney tissues (see **Appendix Supplementary Table 1** for primer sequences).

### 3.5 Morphological analysis of the femur

The length and morphology of the right femur of the 12-week-old rats were documented, focusing on the differences in femur growth among different genotypes of the rats.

### 3.6 Microstructural analysis of the femur

At the age of 12 weeks, the right femur of the rats was scanned by Skyscan-1176 high-resolution *in vivo* Micro-CT (Bruker, Germany) to build a three-dimensional model. Based on this model, the bone volume/total volume (BV/TV), trabecular number (Tb.N), trabecular thickness (Tb.Th), trabecular separation (Tb.Sp), bone mineral density (BMD),cortical bone area (Ct.Ar), ratio of cortical bone area to total tissue area (Ct.Ar/Tt.Ar), cortical bone thickness (Ct.Th) and cortical porosity (Ct.Po) were measured to analyze the differences in the microstructure of femoral metaphysis and diaphysis (cancellous bone and cortical bone) of rats of different genotypes.

### 3.7 Statistical analysis

Analysis of variance (ANOVA) was adopted for multiple group comparisons when the variance was homogeneous; if the *p* value was significant, the Bonferroni multiple test correction was employed for pairwise comparisons. The Kruskal-Wallis test was conducted for multi-group comparisons when the variance was uneven, followed by the Mann-Whitney U test for pairwise comparisons. Statistical significance was set at an alpha level of *p* < 0.05.

## 4 Results

### 4.1 Successful construction of the *PHEX* gene mutation (c.1985delA) rat model

The target site was designed based on the known sequence of the *PHEX* gene (GeneBank ID: 25512) of SD rats. For F1 rats, PCR amplification and agarose gel electrophoresis were performed. Then, gene sequencing was carried out, and the results identified them as F1 heterozygous female rats. After reproduction, genotyping was carried out, resulting in the characterization of five genotypes.

### 4.2 Phenotypes of rats with five different genotypes

#### 4.2.1 Mortality of neonatal rats and body weight and morphology of rats

Weight monitoring and mortality statistics were conducted on newborn rats for a duration of 12 weeks. The mortality rate was found to be higher in *PHEX*-mutant rats compared to wild-type (WT) rats. Whereas, there was no significant difference in the mortality rate observed among the *PHEX*-mutant rats.

Among all genotypes, there was no significant difference in body weight up to 2-3 weeks of age. However, as the rats grew and developed, the weight gain of mutant rats was significantly slower than that of WT rats of the same gender. Additionally, comparing homozygous female rats to heterozygous female rats, the weight gain was slower in the former.

**Figure 3:**
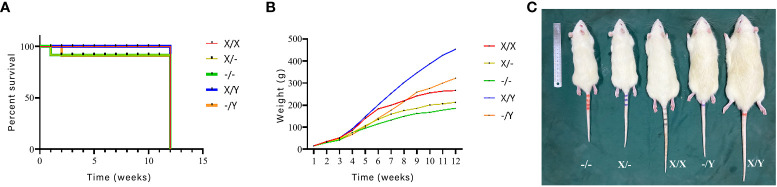
Effects of *PHEX* genotype on mortality of newborn rats as well as body weight and morphology of rats (n=12/group). **(A)** Survival curves of newborn rats; **(B)** Body weights of rats from birth to 12 weeks of age; **(C)** Differences in body morphology of rats.

At the age of 12 weeks, it was observed that mutant rats had significantly smaller body sizes and stubby tails compared to WT rats. Furthermore, homozygous female rats displayed smaller body sizes and stubbier tails when compared to heterozygous females (**Figure 3**).

#### 4.2.2 Serum biochemical parameters in rats

**Figure 4:**
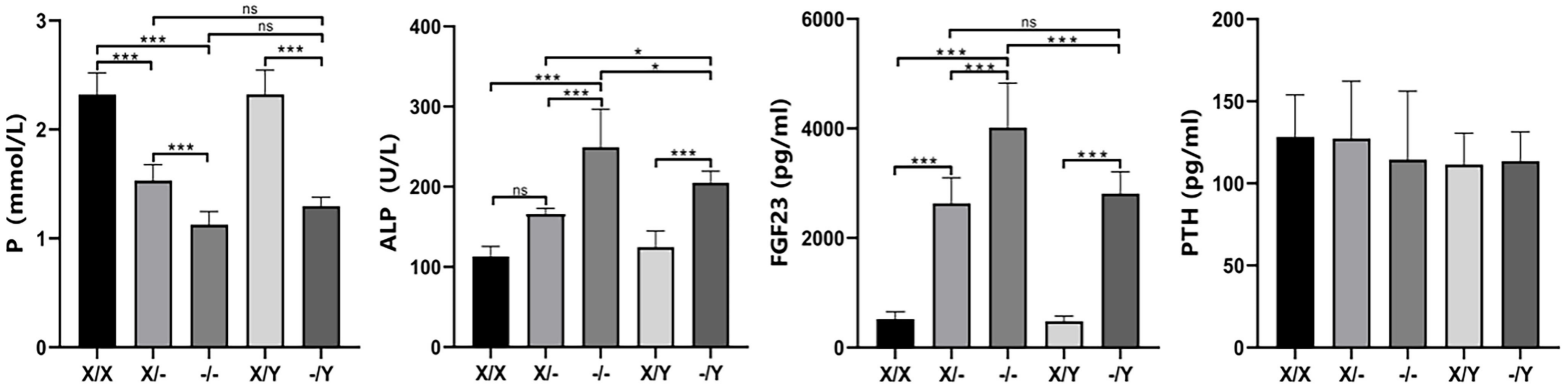
The impact of *PHEX* genotypes on serum biochemical parameters in rats. All values are expressed as mean ± standard deviation; ns: *p* > 0.05, *: *p* < 0.05, ***: *p* < 0.001; For the determination of P and ALP concentrations, X/X: n = 7, X/-: n = 8, -/-: n = 7, X/Y: n = 8, -/Y: n = 7; For the determination of FGF23 and PTH concentrations X/X: n = 7;/-: n = 7, -/-: n = 8, X/Y: n = 8, -/Y: n = 7.

At 12 weeks of age, mutant rats exhibited a significant decrease in phosphorus concentration and an increase in ALP levels compared to WT rats. Homozygous female rats displayed lower phosphorus concentration and higher ALP levels compared to heterozygous female rats. The phosphorus concentration and ALP levels of hemizygous male rats fell between those of homozygous female rats and heterozygous female rats. Noteworthy differences were not observed in calcium, creatinine, and blood urea nitrogen levels among the rats with different genotypes (**Supplementary Figure 3**). ELISA results further demonstrated a substantial increase in FGF23 concentration in mutant rats compared to WT rats. Homozygous female rats exhibited higher FGF23 concentration compared to heterozygous female and hemizygous male rats. The FGF23 concentration of hemizygous male rats was intermediate between that of homozygous female rats and heterozygous female rats. No significant difference was found in PTH concentration among rats with different genotypes (**Figure 4**).

#### 4.2.3 Levels of FGF23, MEPE, and SFRP-4 in rat femoral tissues and levels of K1, Slc34a1, and Slc34a3 in the kidney tissues

Comparing to WT female rats, the expression of *PHEX* was found to be reduced in mutant female rats. Moreover, hemizygous male rats exhibited even lower expression levels than WT male rats. In comparison with WT female rats, WT male rats also displayed lower expression levels of *PHEX*. Additionally, heterozygous female rats showed a decreasing trend in *PHEX* expression relative to WT male rats. Among the mutant rats, homozygous female rats exhibited the lowest expression levels of *PHEX* when compared to heterozygous female rats and hemizygous male rats.

In contrast to WT female rats, the expression of FGF23 was increased in mutant female rats. Likewise, hemizygous male rats displayed higher expression levels of FGF23 compared to WT male rats. Furthermore, homozygous female rats showed higher expression levels of MEPE compared to WT rats, while heterozygous female rats and hemizygous male rats also exhibited higher expression levels of MEPE compared to WT rats. There were no significant differences observed in the expression levels of FGF23 and MEPE among the different genotypes of mutant rats. The expression levels of SFRP-4 were found to be increased in mutant rats compared to WT rats. However, there were no significant differences observed in the expression levels of SFRP-4 among the different genotypes of mutant rats.

**Figure 5:**
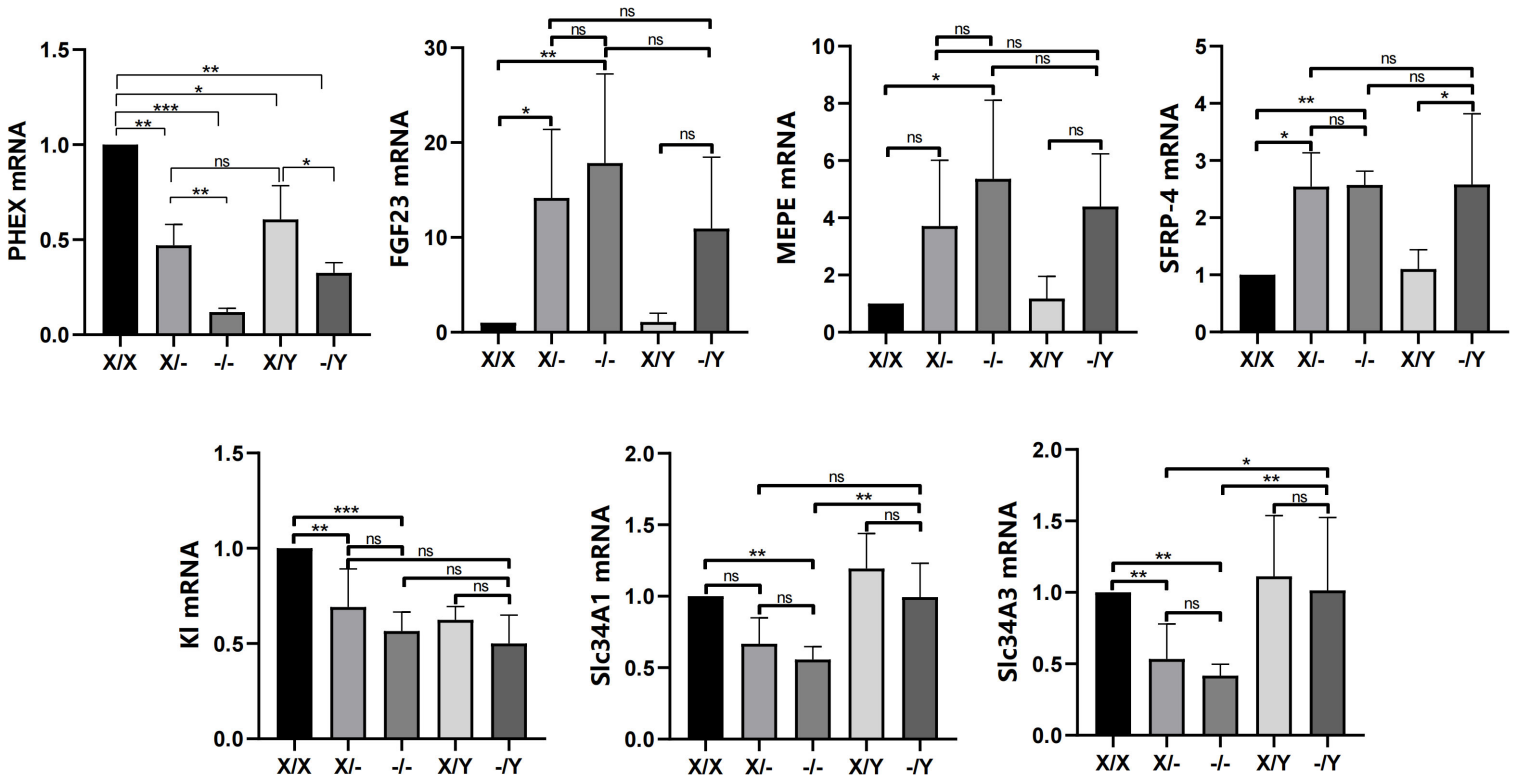
Effects of *PHEX* genotypes on the levels of phosphorus regulators (*PHEX*, FGF23, MEPE and SFRP-4) in the rat femoral tissues and phosphorus metabolic factors (Kl, Scl34A1, and Scl34A3) in the rat kidney tissues. All values are expressed as mean ± standard deviation; ns: *p* > 0.05, *: *p* < 0.05, **: *p* < 0.01, ***: *p* < 0.001; n = 5 in all groups.

Compared to WT rats, mutant female rats showed a decrease in the expression of K1 and Slc34a3, while a decreasing trend was observed in the levels of K1, Slc34a1, and Slc34a3 in hemizygous male rats. In contrast to WT rats, the expression of Slc34a1 was down-regulated in homozygous female rats, and a descending trend was found in both heterozygous female and homozygous male rats. These results further support the observation that there was no significant difference in the expression of K1, Slc34a1, and Slc34a3 among the mutant female rats. Additionally, hemizygous male rats exhibited higher levels of Slc34a1 compared to homozygous female rats, as well as higher levels of Slc34a3 compared to the mutant female rats (**Figure 5**).

#### 4.2.4 Differences in the length and morphology of the rat right femur

**Figure 6:**
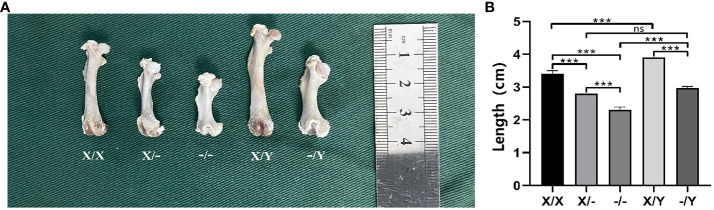
Length and morphology of the rat right femur. **(A)** Morphology of rat femur; **(B)** Length of rat femur; All values are expressed as mean ± standard deviation; ns: *p* > 0.05, ***: *p* < 0.001; X/X: n = 7, X/-: n = 8, -/-: n = 6, X/Y: n = 8, -/Y: n = 7.

The length of the femur was found to be significantly greater in the WT rats compared to the mutant rats of the same gender. The mutant rats exhibited shortened and thickened femoral shafts, along with metaphyseal deformities. Among the female rats, those homozygous for the mutation had even shorter and smaller femurs compared to the heterozygous females, resulting in more pronounced femoral shaft stubbiness and severe metaphyseal deformities (**Figure 6**).

#### 4.2.5 Microstructural differences in cancellous bone in the right femoral metaphysis of rats

**Figure 7:**
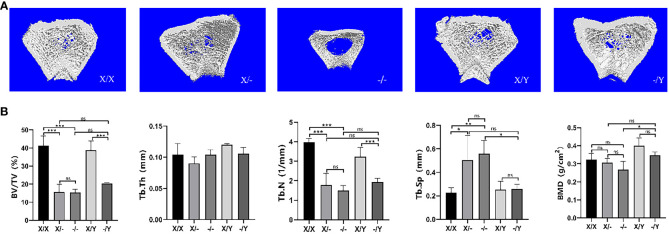
Microstructural differences in cancellous bone in the right femoral metaphysis of rats. **(A)** Three-dimensional image reconstruction of the rat femur; **(B)** Micro-CT scan cancellous bone results of the rat femur. All values are expressed as mean ± standard deviation; ns: *p* > 0.05, *: *p* < 0.05, **: *p* < 0.01, ***: *p* < 0.001; X/X: n = 5, X/-: n = 4, -/-: n = 4, X/Y: n = 4, -/Y: n = 4.

Three-dimensional image reconstruction of the rat right femur revealed that in comparison to WT rats, mutant rats exhibited a decrease in cancellous bone mass and trabecular bone density. No significant difference in bone mass was observed between heterozygous female rats and hemizygous male rats, while homozygous female rats demonstrated a notable reduction in trabecular bone density and the lowest bone mass among the groups (**Figure 7A**).

As compared to the WT rats, BV/TV and Tb.N decreased in the mutant rats, accompanied by a down-regulating trend of BMD and Tb.Th. A significant increase in Tb.Sp (trabecular separation) was observed in mutant female rats compared to WT female rats. However, there was no significant difference in Tb.Sp between hemizygous male rats and WT male rats. Homozygous female rats exhibited decreasing trends in BV/TV, Tb.N, and BMD, while showing increasing trends in Tb.Th and Tb.Sp compared to heterozygous female rats. Hemizygous male rats displayed elevated BV/TV, Tb.N, Tb.Th, and BMD, but reduced Tb.Sp relative to heterozygous female rats. In contrast to homozygous female rats, hemizygous male rats had higher BMD, lower Tb.Sp, and increased BV/TV, Tb.N, and Tb.Th values (**Figure 7B**).

#### 4.2.6 Microstructural differences in cortical bone in the right femoral metaphysis of rats

**Figure 8:**
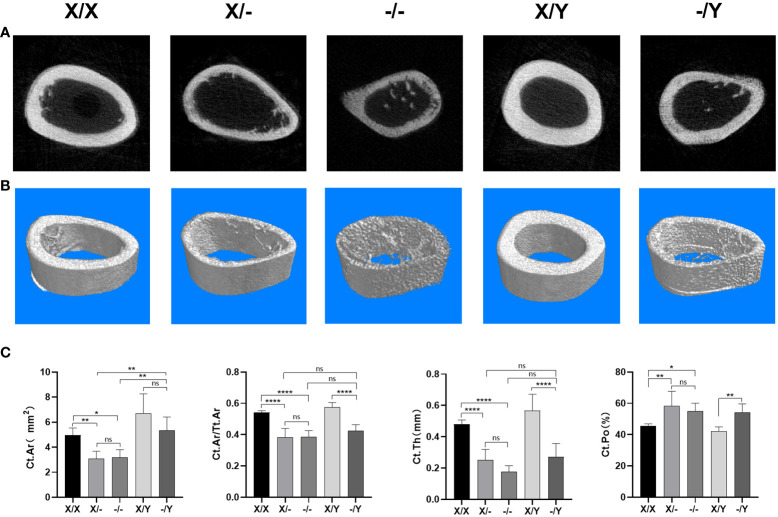
Microstructural differences in cortical bone in the right femoral metaphysis of rats. **(A)** Representative Micro-CT images of the midshaft femoral cortical bone; **(B)** Three-dimensional Cross-sectional image reconstruction of the midshaft femoral cortical bone; **(C)** Micro-CT scan cortical bone results of the rat femur. All values are expressed as mean ± standard deviation; ns: *p* > 0.05, *: *p* < 0.05, **: *p* < 0.01, ***: *p* < 0.001; ***: p < 0.0001; X/X: n = 5, X/-: n = 4, -/-: n = 4, X/Y: n = 4, -/Y: n = 4.

Regarding the femoral cortical bone, three-dimensional image reconstruction revealed that mutant rats exhibited a reduction in cortical bone area compared to WT rats, accompanied by visibly thinner cortical bone thickness (**Figure 8A**). No significant difference in cortical bone area was observed among different mutant rats (**Figure 8B**).

Compared to WT rats, mutant rats displayed a decrease in Ct.Ar and Ct.Ar/Tt.Ar, with no significant variation in Ct.Ar/Tt.Ar observed among the mutant rats. A noticeable increase in Ct.Ar was observed in the female mutant rats compared to male mutant rats. However, there was no significant difference in Ct.Ar between female mutant rats. In terms of Ct.Th and Ct.Po, mutant rats exhibited a significant decrease in Ct.Th and an increase in Ct.Po compared to WT rats. When comparing homozygous female mutant rats to heterozygous female mutant rats, there was a downward trend in Ct.Th. There was no significant difference in Ct.Th and Ct.Po among the mutant rats (**Figure 8C**).

## 5 Discussion

XLH is a metabolic bone disease that is inherited in an X-linked dominant manner. It is primarily caused by the loss of function or mutation of the *PHEX* gene. This genetic abnormality leads to impaired reabsorption of phosphate in the renal tubules, along with increased phosphate excretion. Consequently, affected individuals exhibit impaired mineralization of the epiphysis and cartilage. In this study, we conducted an investigation on two patients diagnosed with rickets and identified a deletion mutation (c.1985delA) in the *PHEX* gene through whole-exome gene sequencing analysis. This deletion mutation caused disruption in the functional activity of the *PHEX* protein. Subsequently, we utilized CRISPR/Cas9-mediated gene editing to successfully generate a rat model of low-phosphate rickets with the same *PHEX* gene mutation (c.1985delA). The purpose of this model was to comprehensively investigate the impact of this frameshift mutation on the development and progression of XLH. It is essential to note that this rat model differs from XLH rat models, such as Hyp and Ska1. Through phenotype analysis, we observed several distinct characteristics in the rat model, including delayed growth and development, a significant decrease in blood phosphorus concentration, an evident increase in FGF23 expression, as well as stubby femurs, femur deformities, and reduced BMD. These observed phenotypes were consistent with the clinical presentations of patients with XLH and previous findings from animal models (15–18).

XLH is inherited in an X-linked dominant manner, with females having two X chromosomes. If either X chromosome carries the pathogenic gene, the disease will occur. Previous studies have indicated that the *PHEX* gene is regulated by a dosage compensation mechanism involving random inactivation of one X chromosome (19). However, the relationship between the severity of clinical phenotypes in XLH patients and the gene dosage associated with the X-linked dominant inheritance pattern has been a subject of intense debate (6–8, 12, 13). Regarding serology, previous research has demonstrated that differences in gene dosage between male and female XLH patients have no impact on biochemical test results (7, 12), even in the first patient known to have a homozygous *PHEX* mutation (20). In our study, we observed that homozygous females with a double *PHEX* gene mutation had the lowest phosphorus levels and the highest concentration of FGF23. A similar pattern was observed for ALP levels. On the other hand, heterozygous females with one normal allele showed a slight improvement in some serum biochemical parameters, such as phosphorus, ALP, and FGF23, but these effects were not statistically significant. It is possible that the *PHEX* mutation gene has reduced the set point of extracellular phosphorus concentration, thereby maintaining phosphorus and FGF23 levels at the desired range. Based on these findings, we propose the hypothesis that the dosage of *PHEX* gene mutation may have an impact on ALP, phosphorus, and FGF23 concentrations.

Studies have indicated that a new mouse model for XLH disease, induced by N-ethyl-N-nitrosourea (ENU) and carrying the *PHEX* ^L222P^ mutation, exhibits an overexpression of *PHEX* transcript and protein, resulting in a loss-of-function phenotype (21). However, our findings in the mutant (c.1985delA) rat model revealed reduced expression of *PHEX* mRNA. We propose that this mutation leads to immature mRNA expression, which is vulnerable to degradation, consequently resulting in decreased expression levels. Regarding phosphorus regulators and metabolic factors, FGF23, released from osteoblasts and osteoclasts, acts on the kidney (22) by binding to the Klotho (Kl):FGF receptor complex (23, 24). This complex inhibits renal phosphate reabsorption and enhances phosphate excretion in the renal tubules by reducing the expression of sodium-driven phosphate cotransporters NaPi-IIa (Slc34a1) and NaPi-IIc (Slc34a3) (25–27). Phosphate regulators, such as MEPE and SFRP-4, play crucial roles in maintaining phosphate homeostasis (28, 29). Overexpression of MEPE and SFRP-4 can lead to abnormal renal phosphate metabolism and bone mineralization, causing hypophosphatemia and increased renal phosphate excretion (30, 31). Previous studies have suggested a potential role of MEPE in the pathogenesis of XLH (32), as elevated levels of MEPE were observed in XLH patients (33). In our study, we found no significant differences in the levels of FGF23, MEPE, SFRP-4, Kl, Slc34a1, and Slc34a3 among the mutant rats. Furthermore, there was no apparent dosage effect, consistent with previous relevant studies (16). Based on our data, it appears that the dosage of the *PHEX* mutation does not significantly influence phosphate homeostasis in bone and kidney tissues.

It was revealed that the presence of a normal *PHEX* allele greatly enhanced bone mineral density levels in female rat with heterozygous mutations (16). In comparison to female patients with heterozygous mutations, male patients with XLH may exhibit more severe bone and dental characteristics (6–8, 13). The presence of two mutant alleles had a significant impact on bone histomorphometry, with homozygous females displaying shorter vertebral body lengths compared to heterozygous females (34). In this study, we observed that double *PHEX* gene mutation in twelve-week-old mutant rats resulted in significantly shorter and smaller femurs. This indicates that the dosage of *PHEX* gene mutation negatively affects bone metabolism and long bone growth. Interestingly, the presence of one normal allele was found to improve the abnormal long bone growth. Further analysis using µCT revealed that *PHEX* gene mutation led to reduced bone mass and trabeculae in cancellous bone, as well as significant thinning of the cortical bone. However, the presence of one normal allele did not have a significant impact on the bone parameters of cancellous and cortical bones.

Our findings suggest that while the extent of abnormal mineral metabolism remains largely unchanged, the presence of one normal allele partially improves the bone’s abnormal mineralization and osteopenia, reducing the severity of intrinsic bone defects in individuals with XLH. Nevertheless, the differences in skeletal parameters are not substantial. Based on these observations, we propose that *PHEX* may have a role in bone mineralization that is independent of phosphate recognition. Alternatively, cells expressing one normal *PHEX* allele might partially rescue the defects present in those lacking *PHEX*.

## 6 Conclusion

A new XLH rat model was successfully developed by CRISPR/Cas9-mediated gene editing, which indicated that the deletion mutation of the *PHEX* gene locus could lead to the initiation of XLH and the model exhibited obvious clinical manifestations of XLH.

Throughout the growth period of female mutant rats, significant the dosage effects were identified in terms of weight gain, body size, tail shape, femur length and morphology, as well as the metabolism of ALP, phosphorus, and FGF23. The presence of one normal allele partially attenuated the severity of the clinical phenotype in XLH. These results highlight the possibility that *PHEX* gene mutations may directly impair bone metabolism through an as-yet-unknown mechanism.

## Data availability statement

The original contributions presented in the study are included in the article/**Supplementary Material**. Further inquiries can be directed to the corresponding authors.

## Ethics statement

The animal study was approved by the lab of animal experimental ethical inspection. All animal study protocols was approved by the ethics committee of Affiliated Hospital of Guangdong Medical University (approval number: AHGDMU-LAC-II (1)-2211-B063). The study was conducted in accordance with the local legislation and institutional requirements. Written informed consent was obtained from the minor(s)’ legal guardian/next of kin for the publication of any potentially identifiable images or data included in this article.

## Author contributions

XC and CC contributed to the conception and design of this study, the performance of experiments, interpretation, data analysis and manuscript writing. SL, FS, CM, JL and WP contributed to the performance of experiments and data analysis. QY, YC, YW and TF performed data analysis and interpretation. XC and GM contributed to the design of this study and acquiring financial support. ML and GM contributed to the data analysis, interpretation and the final approval of the manuscript. All authors contributed to the article and approved the submitted version.
