# Supplementary material for: The contribution of a novel PHEX gene mutation to X-linked hypophosphatemic rickets: a case report and an analysis of the gene mutation dosage effect in a rat model
Source: Front Endocrinol (Lausanne). 2023 Dec 5;14:1251718. doi: 10.3389/fendo.2023.1251718 (PMC10728720; doi:10.3389/fendo.2023.1251718)
Supplement: Supplementary file 1 [file DataSheet_1.pdf]

frontiers

Supplementary Material

The contribution of a novel *PHEX* gene mutation to X-linked hypophosphatemic rickets: a case report and an analysis of the gene mutation dosage effect in a rat model

Xiaoming Chen[1†], Cijing Cai[1,2†], Shaocong Lun[1], Qiuli Ye[4], Weiyuan Pan[1], Yushi Chen[1], Yuexuan Wu[1], Taoshan Feng[1], Faming Su[1], Choudi Ma[1], Jiaxin Luo[1], Meilian Liu[3*] and Guoda Ma[2,5*]

*** Correspondence: Meilian Liu: liumeilian@gdmu.edu.cn ; Guoda Ma: sihan1107@126.com**

1 Supplementary Figures

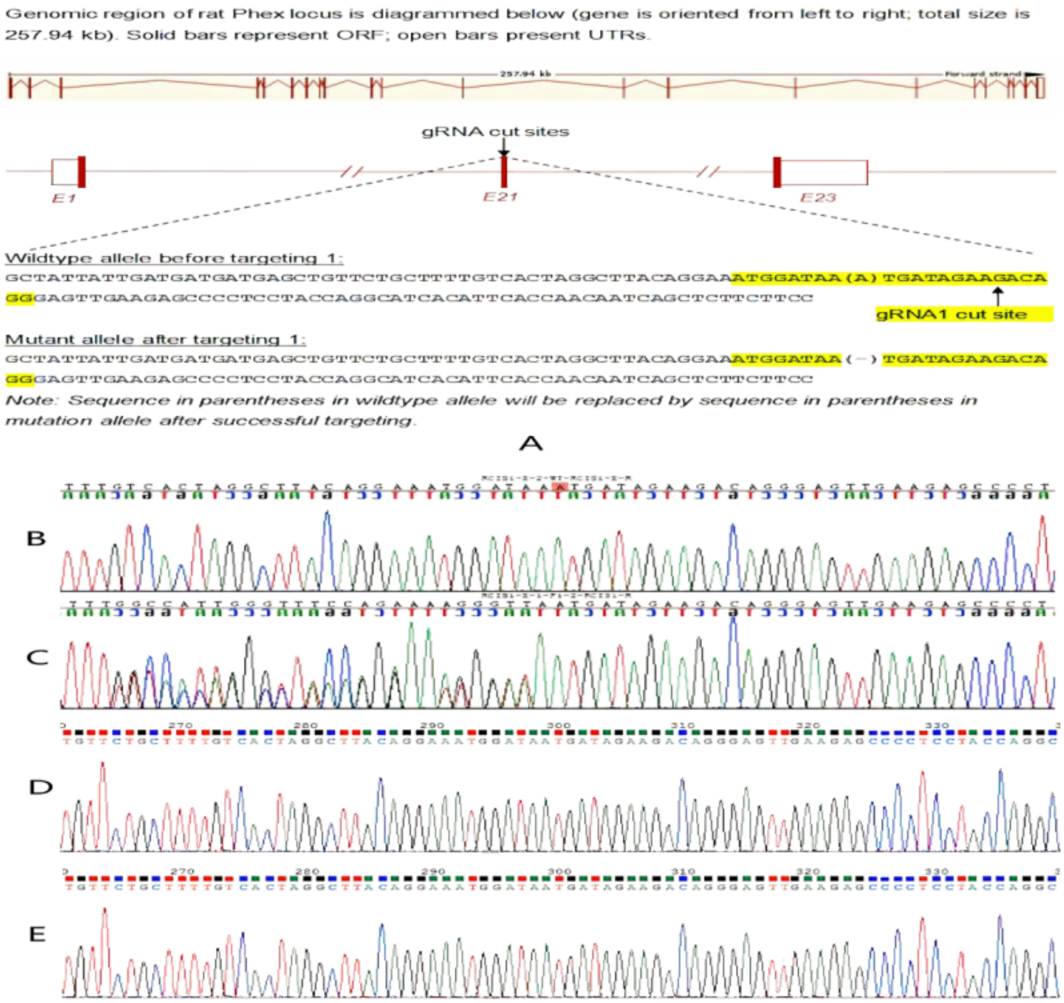

Supplementary Figure 1. A new XLH rat model constructed using CRISPR/Cas9 gene editing technology. A, design of *PHEX* gene-gRNA targeting site; B, Gene sequencing results of WT rats; C,

Gene sequencing results of F1 heterozygous female rats; D, Gene sequencing results of heterozygous female rats; E. Gene sequencing results of homozygous or homozygous male rats.

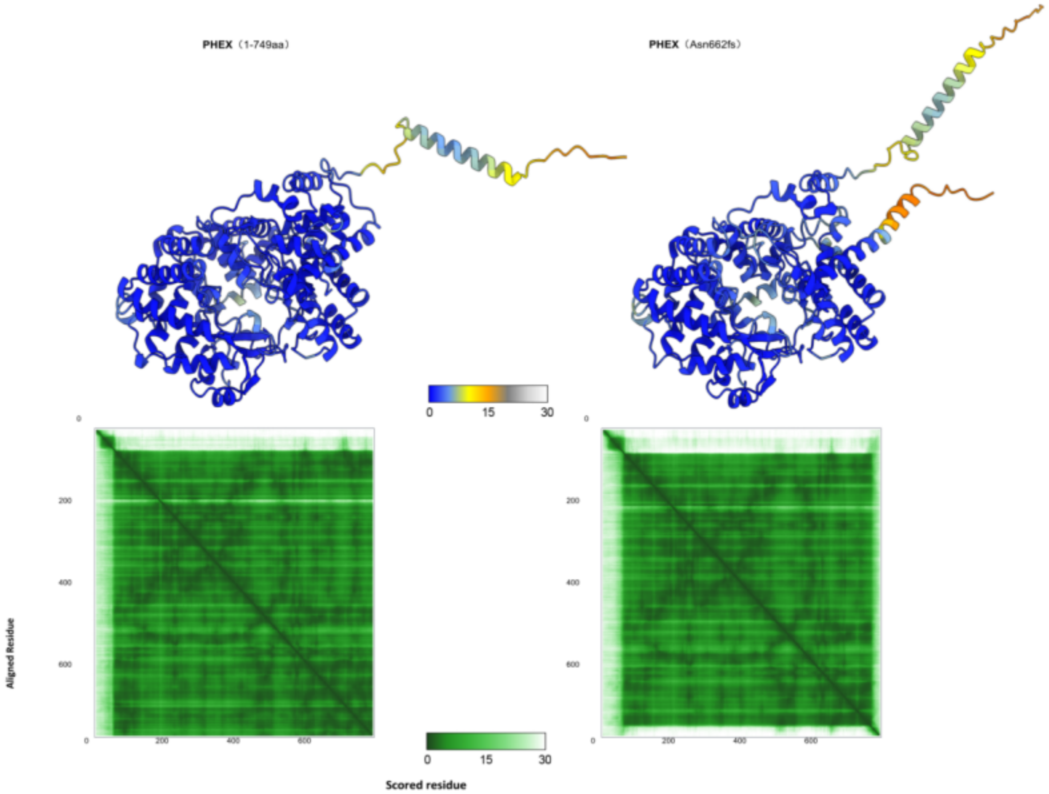

Supplementary Figure 2. The structure prediction analysis of *PHEX* proteins. Per-residue confidence (pLDDT) and Predicted Aligned Error (PAE) for the wild-type and mutant *PHEX* proteins.

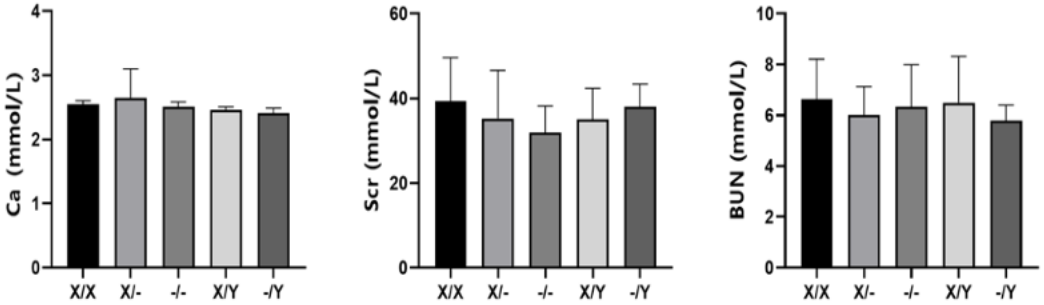

Supplementary Figure 3. A new XLH rat model constructed using CRISPR/Cas9 gene editing technology. A, design of *PHEX* gene-gRNA targeting site; B, Gene sequencing results of WT rats; C, Gene sequencing results of F1 heterozygous female rats; D, Gene sequencing results of heterozygous female rats; E. Gene sequencing results of homozygous or homozygous male rats.

2 Supplementary Tables

Supplementary table 1 Primer sequences of target genes (rat)

Gene	F/R	Primer Sequence(5′ →3′)
GAPDH	F	GACATGCCGCCTGGAGAAAC
	R	AGCCCAGGATGCCCTTTAGT
FGF23	F	CACAGCTACAGCCAGGAACA
	R	GCGGAGATCCATACAAAGGA
MEPE	F	CAGCAGCGGCGGTAACCAAG
	R	CTGTTCTGGTCAAGCAGGTGAAGG
SFRP-4	F	CTATCCCTCGAACGCAAGTC
	R	GGCTGGCTATTTGCTTCTTG
Kl	F	CGTTGAGCCATTACACCACCATCC
	R	GCACCACCGCCACCTGATTG
Slc34a1	F	GCCGTCCTCTACCTCCTCGTG
	R	ATAGCCTGCCAGCCTGCCATAG
Slc34a3	F	TACCAGCAGCATTACCAGCAACAC
	R	AGCCCGAGAGGTCGCATTCC
PHEX	F	TATGGAAGTGGTCCTGCCACAGC
	R	TTGGTCCTGTTTGTCCATTCTAACAG
