# Supplementary material for: The contribution of a novel PHEX gene mutation to X-linked hypophosphatemic rickets: a case report and an analysis of the gene mutation dosage effect in a rat model
Source: Front Endocrinol (Lausanne). 2023 Dec 5;14:1251718. doi: 10.3389/fendo.2023.1251718 (PMC10728720; doi:10.3389/fendo.2023.1251718)
Supplement: Supplementary file 2 [file DataSheet_2.docx]

Supplementary Material

The contribution of a novel *PHEX* gene mutation to X-linked hypophosphatemic rickets: a case report and an analysis of the gene mutation dosage effect in a rat model

Xiaoming Chen^1†^, Cijing Cai^1,2†^, Shaocong Lun^1^, Qiuli Ye^4^, Weiyuan Pan^1^, Yushi Chen^1^, Yuexuan Wu^1^, Taoshan Feng^1^, Faming Su^1^, Choudi Ma^1^, Jiaxin Luo^1^, Meilian Liu^3*^ and Guoda Ma^2,5*^

*** Correspondence:** Meilian Liu: [liumeilian@gdmu.edu.cn ; Guoda Ma: sihan1107@126.com](mailto:liumeilian@gdmu.edu.cn(M.L.),sihan1107@126.com(G.M.))

# Supplementary Figures


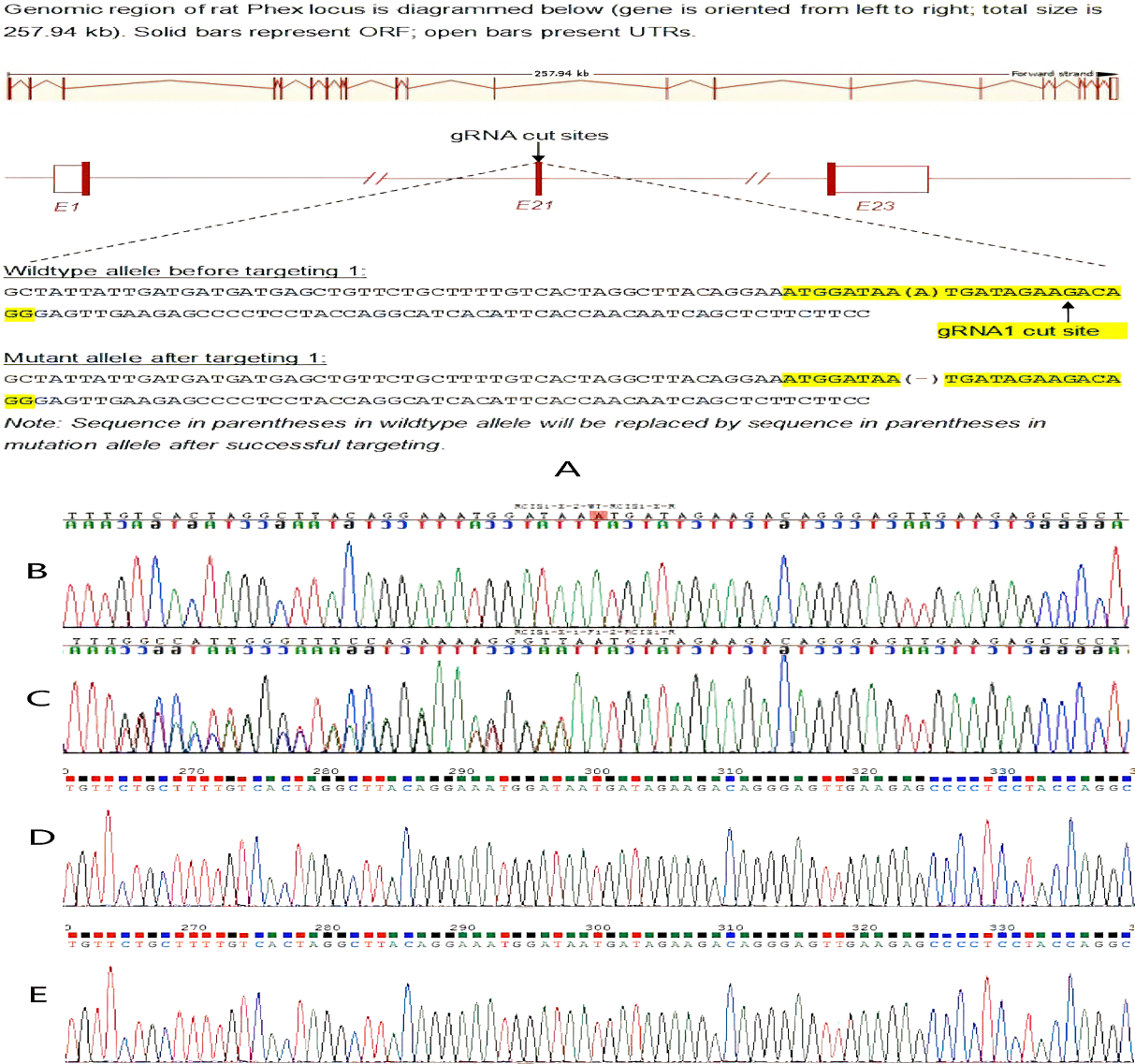


**Supplementary Figure 1.** A new XLH rat model constructed using CRISPR/Cas9 gene editing technology. A, design of *PHEX* gene-gRNA targeting site; B, Gene sequencing results of WT rats; C, Gene sequencing results of F1 heterozygous female rats; D, Gene sequencing results of heterozygous female rats; E. Gene sequencing results of homozygous or homozygous male rats.


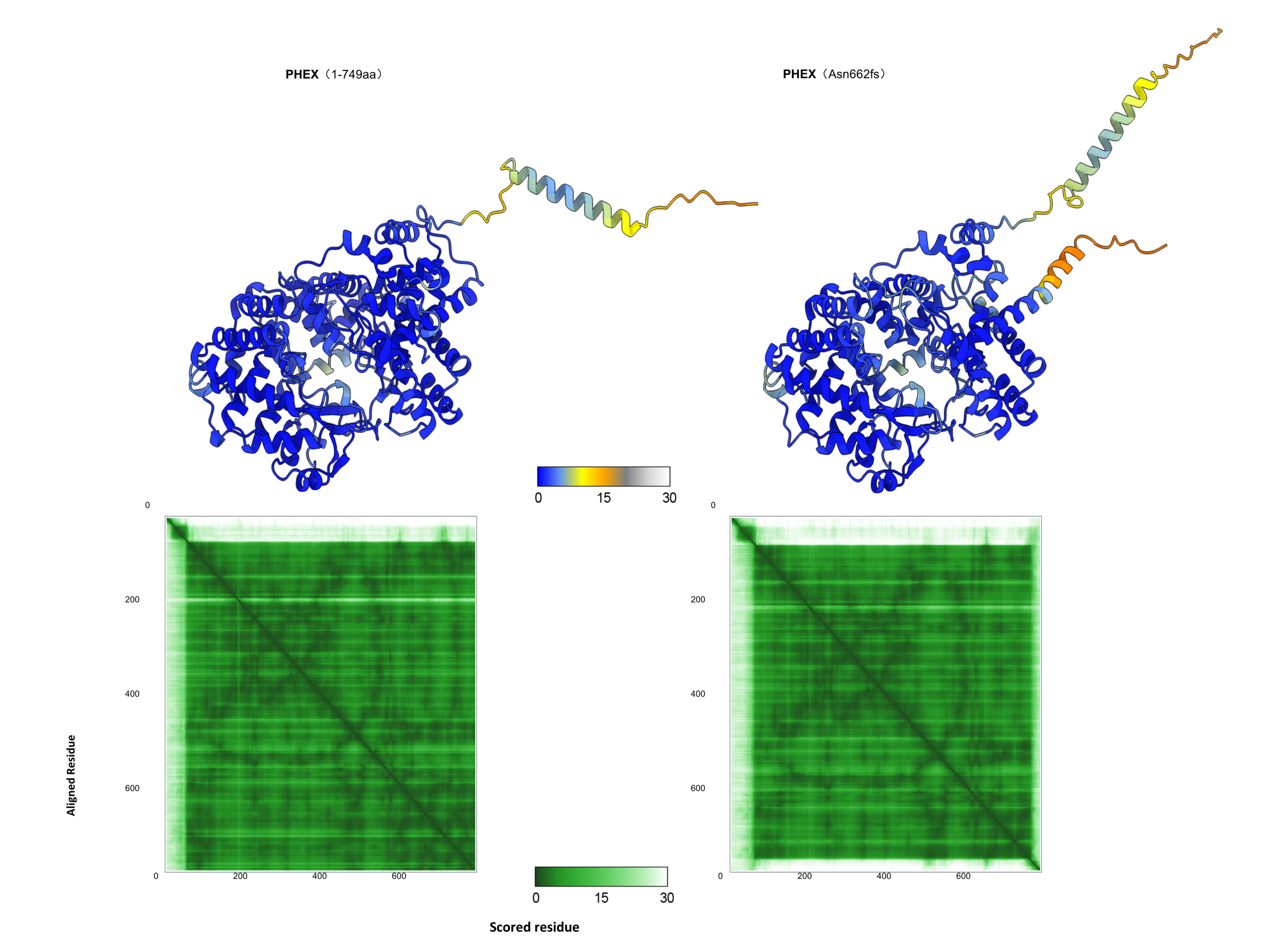


**Supplementary Figure 2.** The structure prediction analysis of *PHEX* proteins. Per-residue confidence (pLDDT) and Predicted Aligned Error (PAE) for the wild-type and mutant *PHEX* proteins.

**
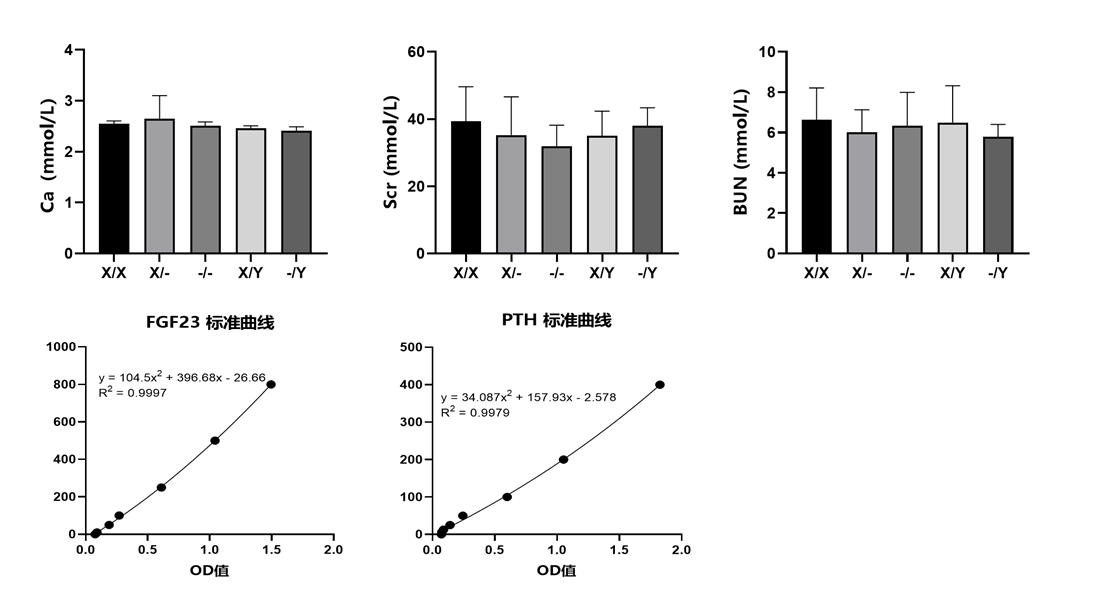
**

**Supplementary Figure 3.** A new XLH rat model constructed using CRISPR/Cas9 gene editing technology. A, design of *PHEX* gene-gRNA targeting site; B, Gene sequencing results of WT rats; C, Gene sequencing results of F1 heterozygous female rats; D, Gene sequencing results of heterozygous female rats; E. Gene sequencing results of homozygous or homozygous male rats.

## 2 Supplementary Tables

**Supplementary table 1** Primer sequences of target genes (rat)

| Gene | F/R | Primer Sequence(5′→3′) |
| --- | --- | --- |
| GAPDH | F | GACATGCCGCCTGGAGAAAC |
|  | R | AGCCCAGGATGCCCTTTAGT |
| FGF23 | F | CACAGCTACAGCCAGGAACA |
|  | R | GCGGAGATCCATACAAAGGA |
| MEPE | F | CAGCAGCGGCGGTAACCAAG |
|  | R | CTGTTCTGGTCAAGCAGGTGAAGG |
| SFRP-4 | F | CTATCCCTCGAACGCAAGTC |
|  | R | GGCTGGCTATTTGCTTCTTG |
| Kl | F | CGTTGAGCCATTACACCACCATCC |
|  | R | GCACCACCGCCACCTGATTG |
| Slc34a1 | F | GCCGTCCTCTACCTCCTCGTG |
|  | R | ATAGCCTGCCAGCCTGCCATAG |
| Slc34a3  PHEX | F | TACCAGCAGCATTACCAGCAACAC |
|  | R  F  R | AGCCCGAGAGGTCGCATTCC  TATGGAAGTGGTCCTGCCACAGC  TTGGTCCTGTTTGTCCATTCTAACAG |
