# Supplementary material for: The contribution of a novel PHEX gene mutation to X-linked hypophosphatemic rickets: a case report and an analysis of the gene mutation dosage effect in a rat model
Source: Front Endocrinol (Lausanne). 2023 Dec 5;14:1251718. doi: 10.3389/fendo.2023.1251718 (PMC10728720; doi:10.3389/fendo.2023.1251718)
Supplement: Supplementary file 3 [file Table_1.docx]

**Supplementary table 1** Primer sequences of target genes (rat)

| Gene | F/R | Primer Sequence(5′→3′) |
| --- | --- | --- |
| GAPDH | F | GACATGCCGCCTGGAGAAAC |
|  | R | AGCCCAGGATGCCCTTTAGT |
| FGF23 | F | CACAGCTACAGCCAGGAACA |
|  | R | GCGGAGATCCATACAAAGGA |
| MEPE | F | CAGCAGCGGCGGTAACCAAG |
|  | R | CTGTTCTGGTCAAGCAGGTGAAGG |
| SFRP-4 | F | CTATCCCTCGAACGCAAGTC |
|  | R | GGCTGGCTATTTGCTTCTTG |
| Kl | F | CGTTGAGCCATTACACCACCATCC |
|  | R | GCACCACCGCCACCTGATTG |
| Slc34a1 | F | GCCGTCCTCTACCTCCTCGTG |
|  | R | ATAGCCTGCCAGCCTGCCATAG |
| Slc34a3  PHEX | F | TACCAGCAGCATTACCAGCAACAC |
|  | R  F  R | AGCCCGAGAGGTCGCATTCC  TATGGAAGTGGTCCTGCCACAGC  TTGGTCCTGTTTGTCCATTCTAACAG |
