# Supplementary figures and images for: The contribution of a novel PHEX gene mutation to X-linked hypophosphatemic rickets: a case report and an analysis of the gene mutation dosage effect in a rat model
Source: Front Endocrinol (Lausanne). 2023 Dec 5;14:1251718. doi: 10.3389/fendo.2023.1251718 (PMC10728720; doi:10.3389/fendo.2023.1251718)

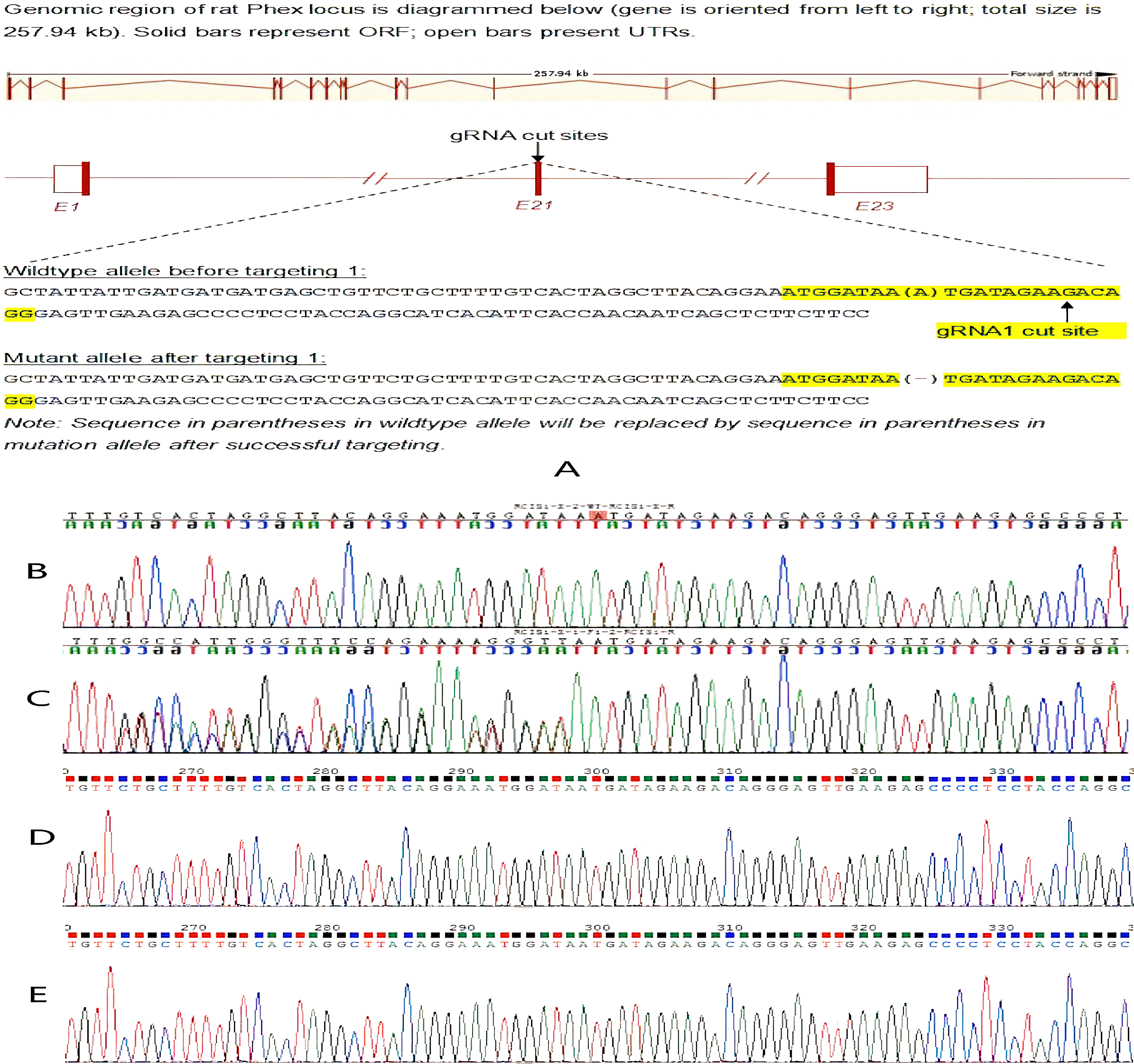

Supplement: Supplementary file 4 [file Image_1.tif]

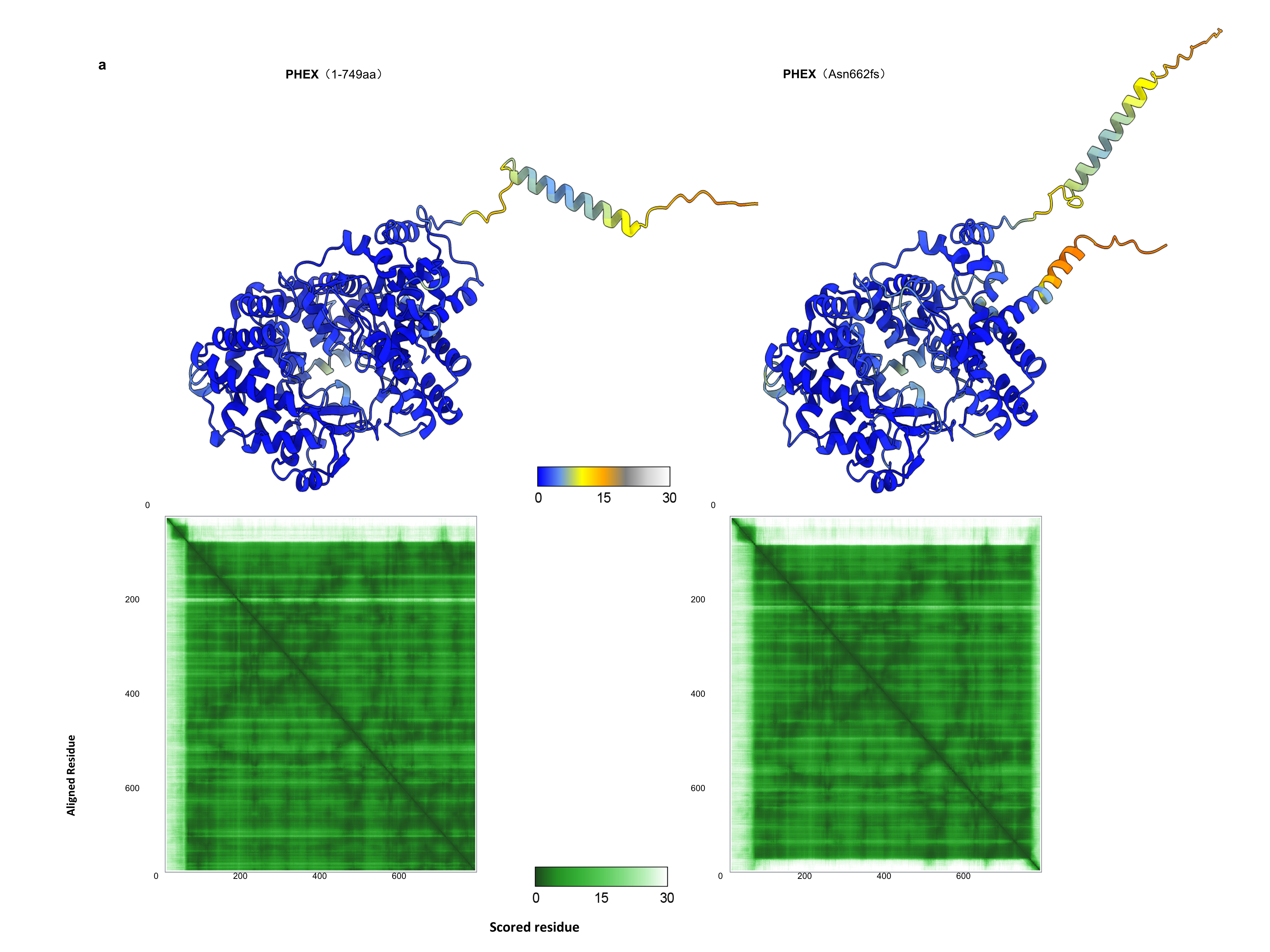

Supplement: Supplementary file 5 [file Image_2.tif]

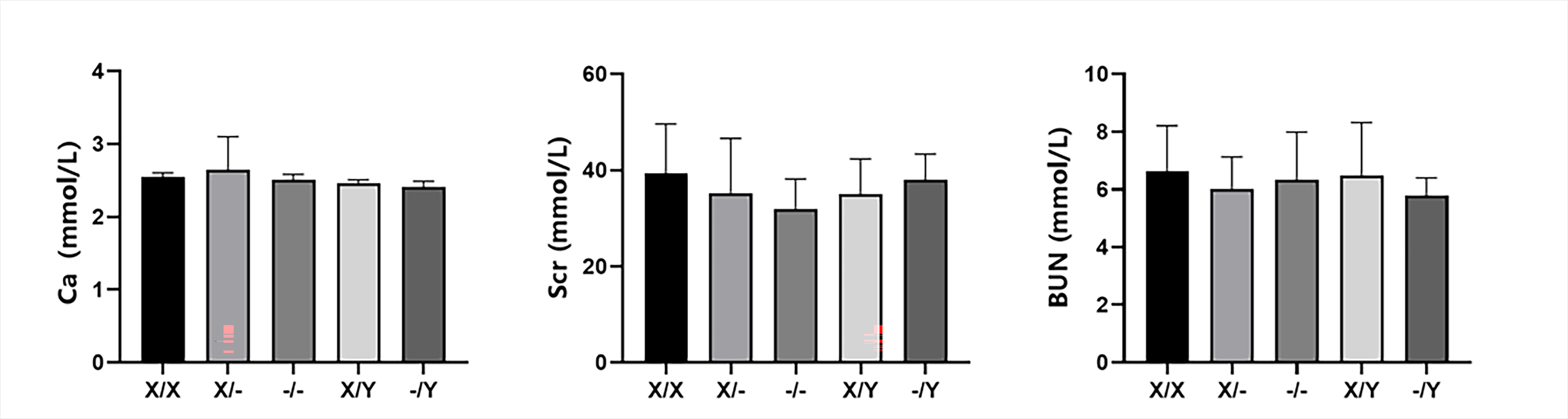

Supplement: Supplementary file 6 [file Image_3.tif]
